# Progressive cephalohematoma in a neonate revealing severe hemophilia a owing to intron 22 inversion: a case report

**DOI:** 10.3389/fped.2025.1649183

**Published:** 2025-07-18

**Authors:** Cheng Peng, Qiuyue Kou, Qianqian Xia, Zhongfen Cao, Lili Liu, Xinlin Hou, Zezhong Tang

**Affiliations:** ^1^ Department of Neonatology, Peking University First Hospital, Beijing, China; ^2^ Department of Pediatrics, The Second Affiliated Hospital of Qiqihar Medical University, Qiqihar, China; ^3^ Department of Neonatology, PKUFH-NINGXIA Women & Children’s Hospital, Yinchuan, China

**Keywords:** hemophilia A, cephalohematoma, neonate, *F8* gene, emicizumab

## Abstract

**Background:**

Hemophilia A is a rare X-linked recessive bleeding disorder characterized by coagulation factor VIII (FVIII) deficiency or dysfunction. While most cases present during early childhood with joint or soft tissue bleeding, neonatal-onset hemophilia A is uncommon and often difficult to diagnose owing to its nonspecific symptoms. Early recognition and a multidisciplinary management approach are critical for preventing life-threatening complications.

**Case Presentation:**

We report a case of a male neonate admitted on day 8 of life with progressive jaundice. Physical examination revealed a large cephalohematoma and multiple skin ecchymomas. Laboratory evaluation revealed anemia and a markedly prolonged activated partial thromboplastin time (APTT). APTT mixing studies indicated factor deficiency, and factor VIII activity was <1%, confirming severe hemophilia A. Genetic analysis identified an intron 22 inversion in the *F8* gene. Initial treatment included fresh frozen plasma, plasma-derived and recombinant factor VIII replacement, and phototherapy. After stabilization, the patient was transitioned to prophylactic emicizumab, which was well tolerated. At 6 weeks of age, the hematoma had nearly resolved with no further bleeding episodes observed.

**Conclusion:**

Early-onset hemophilia A in neonates may present with subtle or atypical symptoms, requiring a high index of suspicion and comprehensive diagnostic evaluation. This case underscores the value of combining functional coagulation assays and molecular testing to confirm diagnosis. It also highlights the potential benefits of early initiation of non-factor prophylaxis. Collaborative cross-disciplinary care is essential to achieve optimal outcomes in neonatal patients with bleeding disorders.

## Introduction

Hemophilia A is a rare X-linked recessive bleeding disorder caused by a deficiency or dysfunction in coagulation factor VIII (FVIII). Traditionally, the prevalence of hemophilia A is estimated to be approximately 1 in 5,000 live male births; however, owing to improvements in diagnostic capabilities and more comprehensive case reporting, the reported prevalence has been steadily increasing (1). According to the 2018 annual report from the European Haemophilia Safety Surveillance (EUHASS) registry, 17,815 individuals were diagnosed with hemophilia A, among whom 7,214 (40.5%) were classified as having severe hemophilia A (2). These data highlight the importance of ongoing diagnosis strategies and monitoring of hemophilia A, and emphasize the importance of early diagnosis and appropriate management to improve long-term health outcomes.

Although hemophilia A typically presents in early childhood with spontaneous joint or soft tissue bleeding, neonatal-onset cases are uncommon and present diagnostic and therapeutic challenges owing to their nonspecific symptoms and the limited ability of neonates to express discomfort.

Neonates with severe hemophilia A may initially present with atypical bleeding signs, such as cephalohematoma, prolonged bleeding from venipuncture sites or heel pricks during medical procedures, unexplained anemia, and soft tissue bleeding. Mucocutaneous (e.g., oral and nasal) and extracranial bleeding are also common (3, 4). These early bleeding manifestations differ from the classic hemarthrosis observed in older children, making early recognition and diagnosis crucial for preventing life-threatening hemorrhagic complications.

This case report describes a male neonate with severe hemophilia A, who was first diagnosed during the neonatal period following the development of progressive jaundice, significant cephalohematoma, and prolonged activated partial thromboplastin time (APTT). Unlike typical pediatric presentations, this early onset case required prompt diagnostic evaluation and initiation of factor VIII replacement therapy. Genetic testing confirmed an intron 22 inversion in the *F8* gene, a mutation strongly associated with severe disease. Early prophylactic management, including the use of emicizumab post-discharge, highlights the importance of individualized therapy and regular monitoring to achieve favorable developmental outcomes and prevent recurrent bleeding episodes.

## Case description

A male neonate was admitted to our hospital on day 8 of life with progressive jaundice which had been first noted on day 2 of life and had worsened over time. At presentation on day 8, transcutaneous bilirubin was 17 mg/dl, and serum total bilirubin was 316.2 μmol/L with direct bilirubin of 11.5 μmol/L. The infant was alert, feeding well, and exhibited no signs of acute bilirubin encephalopathy (e.g., poor feeding, lethargy, or high-pitched cry).

The patient was the first child of nonconsanguineous parents born at 39^+4^ weeks of gestation via cesarean section. His birth weight was 3,800 g and his Apgar scores were 10 at 1, 5, and 10 min. No perinatal complications occurred. Owing to significant hyperbilirubinemia, the patient was admitted for further evaluation.

**Figure 1:**
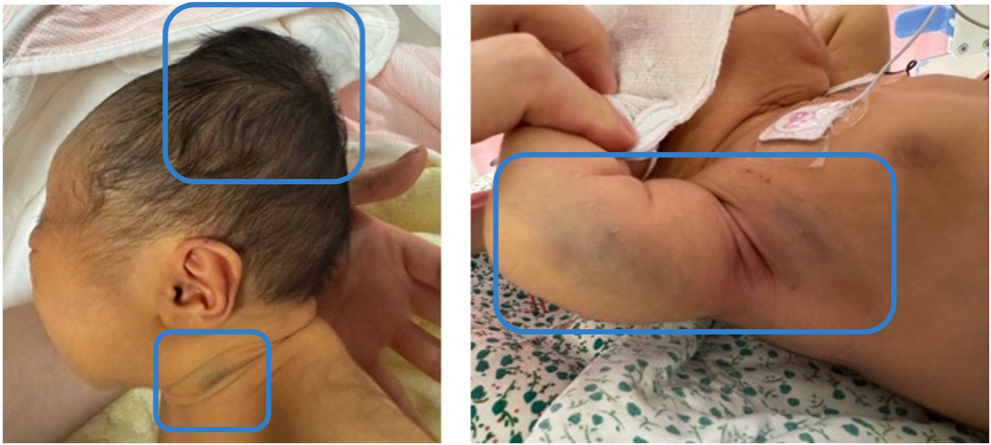
Physical examination shows a cephalohematoma and multiple skin ecchymoses (highlighted within the blue boxes).

Physical examination revealed that the neonate was alert and had generalized jaundice. A sizable cephalohematoma (approximately 6 × 10 cm), which was tense and fluctuating, was noted over the parietal scalp. Bruising was observed at the venipuncture sites on the neck and right upper arm (Figure 1). Joint swelling was not observed. Neurological, cardiac, respiratory, and abdominal examination results were unremarkable. The neonatal behavioral neurological assessment and bilirubin-induced neurologic dysfunction scores were 39 and 0, respectively.

**Figure 2:**
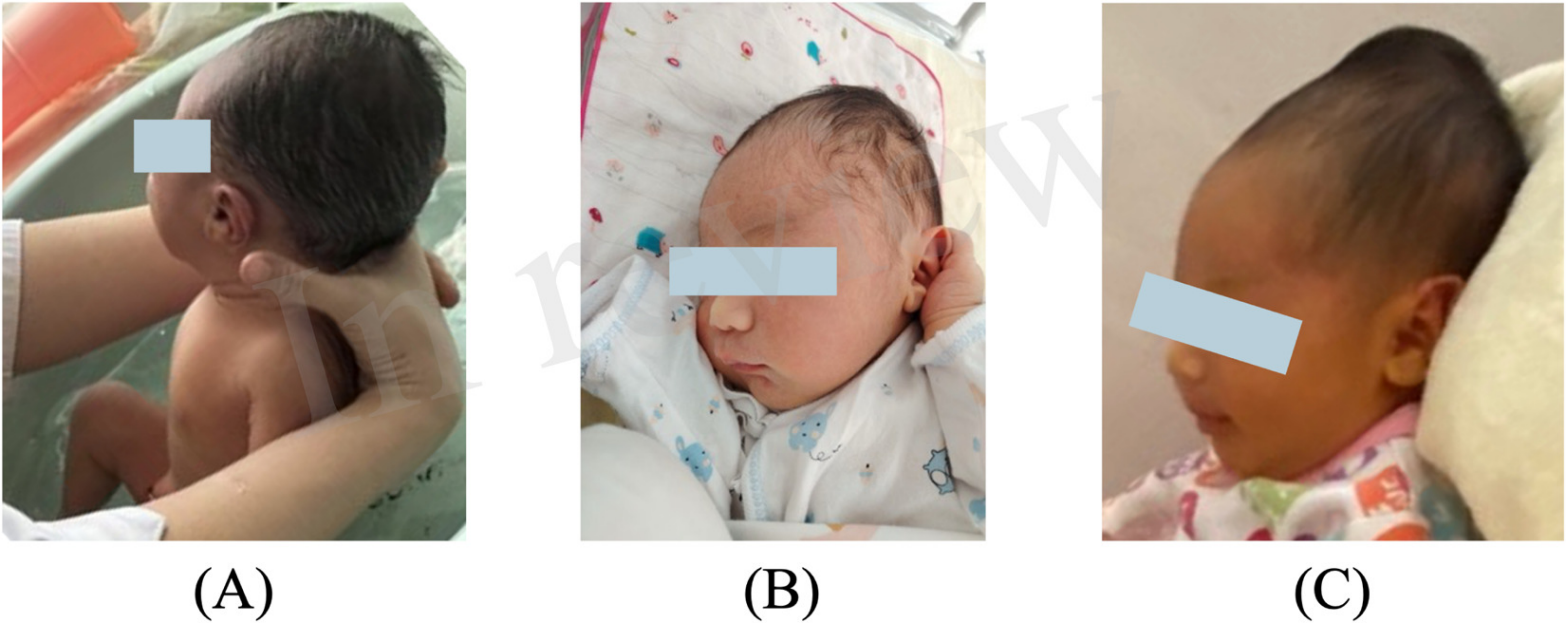
Progressive enlargement of the cephalohematoma on **(A)** day 3, **(B)** day 4, and **(C)** day 8 (the day of admission).

After admission, the progressive enlargement of the cephalohematoma raised concerns about an underlying coagulation dysfunction. Detailed history-taking revealed that the cephalohematoma had increased steadily since birth, (Figure 2) prompting further coagulation function tests to better understand the underlying conditions and guide our treatment approach.

Laboratory workup upon admission revealed anemia (hemoglobin level, 86 g/L). Coagulation tests revealed a significantly prolonged APTT (180.6 s). The patient's factor VIII activity was <1%, with normal levels of other clotting factors, confirming the diagnosis of severe hemophilia A.

The APTT mixing test was performed 2 days after admission. The results were as follows: APTT1:182 s, APTT2:31.5 s, APTT3:36.3 s, APTT4:184.9 s, APTT5:33.7 s, APTT6:38.7 s, APTT7:39.6 s; Rosner Index: 2.6% (Table 1). These results demonstrated that the prolonged APTT was corrected upon mixing with normal plasma, suggesting a factor deficiency rather than the presence of a clotting inhibitor. This further confirmed the diagnosis of hemophilia A. Neuroimaging (cranial ultrasound and CT) revealed bilateral parietal bone rarefaction and a scalp hematoma without evidence of acute intracranial hemorrhage. Additional imaging findings (cardiac, abdominal, and soft-tissue ultrasonography) were largely unremarkable.

**Table 1:** The APTT mixing test.

Postnatal age	APTT1	APTT2	APTT3	APTT4	APTT5	APTT6	APTT7	Rosner index
Day 9	182 s	31.5 s	36.3 s	184.9 s	33.7 s	38.7 s	39.6 s	2.6%

**Table 2:** Treatment and dosage schedule during hospitalization.

Postnatal age	Treatment	Dosage
Day 9	Plasma + Red blood cell transfusion	20 ml/kg respectively
Day 10	Human plasma-derived factor VIII	145 IU (36 IU/kg)
Day 17	Recombinant factor VIII	190 IU (46 IU/kg)
Day 24	Recombinant factor VIII	200 IU (48 IU/kg)

**Figure 3:**
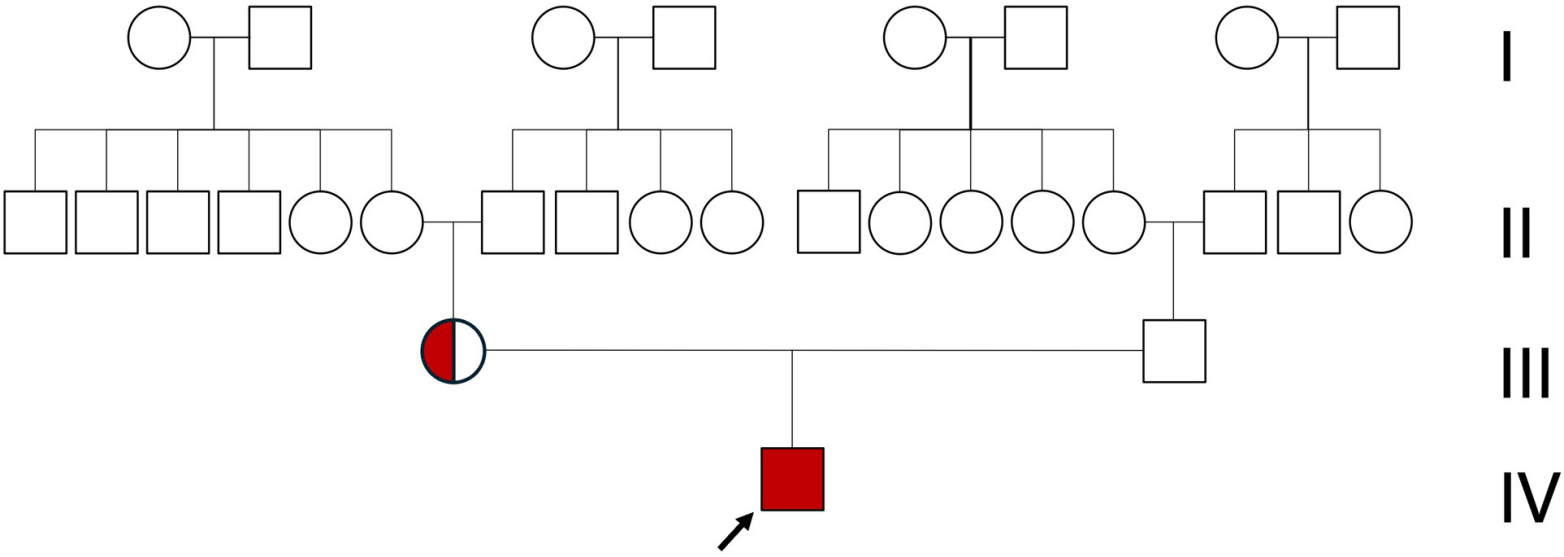
Family pedigree of the patient (black arrow). Only the patient is affected by hemophilia, while all other family members show no clinical signs. The mother (circle) is a carrier of hemophilia.

Genetic testing revealed an intron 22 inversion in the *F8* gene, which led to the diagnosis of severe hemophilia A. Maternal genetic analysis confirmed that the variant was inherited, with the mother identified as a heterozygous carrier (Figure 3). At present, only the parents underwent genetic and clinical evaluation. Information on the extended family was obtained through detailed interviews, which revealed no known history of bleeding disorders. It is speculated that the intron 22 inversion in this case may have been inherited from the maternal grandmother. The mutation may, in turn, have been transmitted to her from the maternal great-grandmother, consistent with the X-linked recessive inheritance pattern of hemophilia A. However, due to privacy considerations, genetic testing was not extended to other family members. It is also plausible that the maternal grandmother's sister could be the carrier of the *F8* gene mutation; however, there is no available clinical or genetic information regarding potential manifestations in their descendants.

Consequently, the infant was diagnosed with severe hemophilia A, neonatal hyperbilirubinemia, cephalohematoma, neonatal anemia, and skin ecchymosis. The treatment included a transfusion of fresh frozen plasma and red blood cell on day 9, followed by the administration of human plasma-derived factor VIII concentrate (145 IU, 36 IU/kg) on day 10. This was followed by recombinant factor VIII infusion (190 IU, 46 IU/kg) on day 17. Intermittent phototherapy was applied to manage the jaundice. The infant was discharged 21 days after birth with substantial resolution of the cephalohematoma, stable condition, and feeding well. To ensure the safety of the infant, the patient was readmitted for a second hospitalization, during which a recombinant factor VIII infusion was administered (200 IU, 48 IU/kg) on day 24 (Table 2). At 6 weeks of age, the hematoma had nearly resolved with no new bleeding episodes and the growth and development were age-appropriate. After discharge, the patient was started on emicizumab prophylaxis (3 mg/kg administered weekly for a total of four doses) with long-term prophylactic dosing adjusted based on serum concentration monitoring.

## Discussion

This case raises an important clinical question: what does it mean when a seemingly benign cephalohematoma continues to grow after birth? Cephalohematomas are commonly observed in neonates, particularly after instrument-assisted or prolonged deliveries, and are generally considered self-limiting lesions that resolve spontaneously within 1–6 weeks without the need for medical intervention (5). Hyperbilirubinemia often accompanies cephalohematoma because of red blood cell breakdown, and this combination is frequently encountered in clinical practice (6). As a result, clinicians may assume a benign clinical course, potentially delaying recognition of underlying coagulopathies or other pathological conditions.

In the present case, the infant's cephalohematoma was noted during the rooming-in period and was initially interpreted as a typical postnatal finding. The accompanying jaundice was considered clinically explainable and the parents were advised to monitor the hematoma at home. However, instead of stabilizing, the cephalohematoma progressively enlarged after discharge, an abnormal pattern not initially recognized by the parents. Progressive enlargement in the absence of additional trauma should prompt clinicians to investigate the possibility of underlying bleeding disorders. The differential diagnoses in such cases include thrombocytopenia, vitamin K deficiency bleeding, disseminated intravascular coagulation, and congenital coagulation factor deficiencies such as hemophilia. In this case, the unusual progressive enlargement of the cephalohematoma raised clinical concerns and led to further evaluation, ultimately confirming the diagnosis of severe hemophilia A.

This case also highlights the evolving role of traditional coagulation tests, such as the APTT mixing study, in an era in which genetic and factor assays are increasingly available.

With advances in molecular diagnostics and quantitative assays for coagulation factor activity, clinicians are now equipped with more precise tools to diagnose inherited bleeding disorders. In this case, factor VIII activity was found to be <1%, and subsequent genetic testing identified an intron 22 inversion in the *F8* gene—findings that definitively confirmed the diagnosis of severe hemophilia A.

However, it is important to recognize that genetic testing typically requires at least 2 weeks to yield results. Delayed diagnostic confirmation can significantly increase the risk of morbidity and mortality in critically ill neonates who present with unexplained bleeding or rapidly enlarging hematomas. Timely diagnosis is essential for guiding factor replacement therapy and preventing potentially life-threatening complications.

In contrast, the APTT mixing study can be performed rapidly and remains a useful functional assay during the initial diagnostic workup. It helps distinguish between factor deficiencies and the presence of circulating inhibitors, which is crucial for immediate clinical decision-making (7, 8). In resource-limited settings or in cases of diagnostic uncertainty, the mixing study serves as a practical and informative tool, especially when used in conjunction with basic coagulation screening.

Therefore, despite the increasing reliance on advanced laboratory technologies, APTT mixing studies are clinically relevant. Rather than being replaced, APTT mixing should be considered a complementary diagnostic step, particularly in urgent or diagnostically ambiguous presentations. This case reinforces the importance of integrating both modern and traditional approaches to ensure timely and accurate diagnosis of neonatal bleeding disorders.

In parallel, genetic testing plays a crucial role in confirming the definitive diagnosis and characterizing the underlying mutation. In this patient, molecular analysis identified an intron 22 inversion in the *F8* gene—the most common causative mutation associated with severe hemophilia A (9). Such information not only confirms the diagnosis at the genetic level, but also informs clinical severity, guides treatment planning, and enables cascade testing and genetic counseling for at-risk family members.

Following diagnosis, the patient received coagulation support with human plasma-derived factor VIII concentrate on day 10 of life owing to the unavailability of recombinant factor VIII and the need to provide prompt hemostatic coverage to ensure patient safety. Although plasma-derived factor VIII remains an effective option, its use necessitates careful consideration of potential risks, including the theoretical transmission of blood-borne infectious agents, despite rigorous donor screening and viral inactivation processes (10). This underscores the importance of timely access to readily available recombinant products, particularly in pediatric and neonatal emergency settings.

Repeated administration of recombinant factor VIII is associated with the well-documented risk of inhibitor development. The presence of inhibitors is one of the most serious and challenging complications in the clinical management of hemophilia because it compromises the efficacy of standard factor replacement therapy and significantly increases the risk of uncontrolled bleeding (11, 12). In response to these challenges, the emergence of novel therapeutic agents has expanded the options for both prophylaxis and treatment of bleeding episodes in patients treated with inhibitors, offering new hope for improved clinical outcomes in this high-risk population.

Emicizumab has emerged as an optimal first-line prophylactic agent in settings where resources and regulatory approval are available. Its unique mechanism of action, subcutaneous route of administration, long half-life, and ability to maintain consistent therapeutic levels make it particularly suitable for use in neonates. Moreover, as a nonfactor therapy, emicizumab does not induce formation of factor VIII inhibitors, providing a distinct immunological advantage for early prophylaxis. In this case, the timely transition to emicizumab following initial stabilization allowed for effective bleeding prevention and prevented further exposure to factor VIII during the high-risk period for inhibitor development.

Emicizumab is a bispecific monoclonal antibody that mimics the function of activated factor VIII by bridging activated factors IX and X (13). It offers several advantages over conventional factor VIII replacement therapy, particularly in neonates and infants. Unlike recombinant factor VIII, emicizumab is administered subcutaneously, which greatly reduces the need for frequent intravenous access. This is a considerable benefit for this age group as venous access is often difficult and distressing. Moreover, emicizumab has a long half-life, allowing for weekly or biweekly dosing and maintenance of steady therapeutic levels, reducing the peaks and troughs observed with factor infusions. Importantly, emicizumab is effective in patients with and without factor VIII inhibitors, making it a versatile option for prophylaxis in severe hemophilia A (14, 15). Given its favorable safety profile, ease of administration, and efficacy in preventing spontaneous bleeding, emicizumab represents a major advancement in the long-term management of severe hemophilia A, especially in neonates, where early sustained prophylaxis is critical for preventing early joint disease and intracranial hemorrhage. Although emicizumab has been approved for prophylactic use in patients with hemophilia A across various age groups, pharmacokinetic data specific to neonates are limited. This poses a challenge when determining the appropriate dosing regimens for this population. The currently recommended regimen includes an initial loading dose of 3 mg/kg once weekly for 4 weeks, followed by a maintenance dose of 1.5 mg/kg weekly (16).

Building upon the success of emicizumab, new non-factor therapies with distinct mechanisms of action are currently under investigation. With the continuous development of nonfactor therapies, marstacimab has emerged as a promising investigational agent for the management of hemophilia A and B, including patients with inhibitors. Marstacimab is a fully human monoclonal antibody that targets tissue factor pathway inhibitors, thereby enhancing thrombin generation through the extrinsic coagulation pathway (17, 18). This mechanism is distinct from that of emicizumab and provides an alternative approach for restoring the hemostatic balance in patients with hemophilia. Although marstacimab holds potential for use in pediatric and neonatal care, its clinical implementation in these vulnerable populations will depend on the results of future studies confirming its safety, pharmacokinetics, and long-term efficacy.

## Conclusion

This case highlights the importance of early recognition and comprehensive evaluation of unexplained progressive cephalohematomas in neonates. Timely diagnosis of severe hemophilia A was achieved by integrating conventional coagulation tests, APTT mixing studies, and genetic analysis. The successful outcomes also underscore the critical role of multidisciplinary collaboration, particularly among neonatology, pediatric hematology, and radiology, in ensuring accurate diagnosis and appropriate management. The patient benefited from early replacement therapy and prophylactic emicizumab, reflecting the growing role of nonfactor therapies in neonatal hemophilia care.

## Data Availability

The original contributions presented in the study are included in the article/Supplementary Material, further inquiries can be directed to the corresponding author.
